# Enhanced liver fibrosis score is associated with cognitive performance during an 11-year follow-up period

**DOI:** 10.1097/HC9.0000000000001059

**Published:** 2026-10-19

**Authors:** Emilia Vataja, Katri Sääksjärvi, Jenni Lehtisalo, Iris Erlund, Terhi Vihervaara, Tiia Ngandu, Seppo Koskinen, Fredrik Åberg, Antti Jula, Juha Rinne, Miikka-Juhani Honka, Laura Ekblad

**Affiliations:** 1 Department of Internal Medicine, University of Turku, Turku, Finland; 2 Finnish Institute for Health and Welfare, Helsinki, Finland; 3 University of Eastern Finland, Kuopio, Finland; 4 Institute for Nutrition and Health Research, Helsinki, Finland; 5 Department of Transplantation and Liver Surgery, Helsinki University Hospital and University of Helsinki, Helsinki, Finland; 6 Department of Neurology, Wellbeing Services County of Southwestern Finland, Turku, Finland; 7 Turku PET Centre, University Hospital of Turku, Turku, Finland; 8 Turku PET Centre, University of Turku, Turku, Finland; 9 Institute of Clinical Physiology, National Research Council (CNR), Pisa, Italy; 10 Department of Geriatrics, Wellbeing Services County of Southwestern Finland, Turku, Finland

**Keywords:** cognition, ELF, epidemiology, liver fibrosis, MASLD

## Abstract

**Introduction:**

Metabolic dysfunction–associated steatotic liver disease (MASLD) has been associated with poorer cognitive performance and greater cognitive decline. However, the liver–brain axis and the role of liver health in cognitive decline are still poorly understood. Thus, using both cross-sectional and longitudinal designs, we aimed to study how liver fibrosis score (ELF), an indicator of liver fibrosis, is associated with cognitive performance.

**Methods:**

This study was based on the Health 2000 Survey and its follow-up, the Health 2011 survey, which collected data from a nationally representative sample of the Finnish adult population. At baseline, verbal fluency, wordlist learning, delayed wordlist recall, and simple and multiple-choice reaction time tests were conducted. At follow-up, all tests except the reaction time tests were repeated. A global z-score was derived from the individual cognitive domains. Multivariable regression analysis was performed to study the association between ELF and cognitive performance while adjusting for demographic, metabolic, and cardiovascular covariates.

**Results:**

Cross-sectionally (n=5115; mean age 53.2 y; 58.6% women), high ELF was associated with poorer global cognition and slower simple and multiple-choice reaction times compared with low ELF. High ELF was also associated with worse global cognition and performance in the wordlist learning test, both cross-sectionally and longitudinally (n=2490; mean age 48.7 y at baseline; 58.0% women).

**Discussion:**

High ELF, an indicator of liver fibrosis, was associated with poorer global cognition both cross-sectionally and longitudinally. Worse performance was observed in cognitive domains that reflect poorer processing speed and executive function, verbal learning, attention, and working memory. These results imply that liver fibrosis could represent an early modifiable marker of cognitive vulnerability.

## INTRODUCTION

Liver fibrosis is a condition where liver tissue is substituted by fibrous connective tissue, often due to repeated or persistent insults. Metabolic dysfunction–associated steatotic liver disease (MASLD) is the most common cause of liver fibrosis.^1^ Less common conditions that can lead to fibrosis of the liver include, for example, chronic viral hepatitis (B and C), excessive alcohol consumption, autoimmune hepatitis, and primary biliary cholangitis.^2^

MASLD is a prevalent liver manifestation of obesity, type 2 diabetes mellitus (DM), and metabolic syndrome, although MASLD also occurs in the lean population.^3^ MASLD covers the continuum from simple steatosis to fibrosis that can lead to cirrhosis, and sometimes even to hepatocellular carcinoma.^3^ Steatosis can lead to fibrosis and cirrhosis via steatohepatitis, where persisting inflammation slowly replaces functional liver parenchyma with fibrotic tissue.^4^

Until recently, there has been no pharmacological treatment for MASLD. Treatments targeting insulin resistance (IR) or weight loss are often beneficial, and weight loss may lead to the resolution of simple steatosis or steatohepatitis.^5^ Furthermore, acetylsalicylic acid has been shown to reduce hepatic fat content,^6^ and sodium-glucose co-transporter-2 (SGLT-2) inhibitors have been associated with reduced all-cause mortality and hepatic decompensation in patients with MASLD steatohepatitis and DM.^7^

For measuring liver fibrosis, many non-invasive methods are available in research and clinical settings. Elastography can be used to measure the stiffness of the liver parenchyma, but its availability is limited to larger healthcare centers. In contrast, scoring systems are more readily available in primary healthcare and are of importance in screening for liver disease. In this study, we focus on the enhanced liver fibrosis score (ELF). It has been shown to be useful in predicting risk for liver-related hospitalizations, cancer, liver decompensation, and mortality.^8^,^9^

Recent research has shown MASLD to be associated with cognitive dysfunction and cognitive decline.^3^,^10^,^11^,^12^,^13^,^14^,^15^,^16^,^17^,^18^ However, no studies have examined the performance of ELF in predicting cognitive decline. Furthermore, MASLD is difficult to diagnose in primary healthcare, and thus, it is of interest how the readily available ELF associates with cognitive dysfunction. Here, we used a nationally representative sample to study whether the presence of fibrosis, measured by ELF, is associated with poorer cognitive performance cross-sectionally and longitudinally during an 11-year follow-up period.

## METHODS

### Study design and participants

The Health 2000 and 2011 surveys were conducted in 2000–2001 and 2011–2012, respectively, by the Finnish Institute for Health and Welfare. The Health 2000 survey comprises a nationally representative sample of the Finnish adult population (n=8028).^19^ Every participant from the Health 2000 survey was invited to take part in the Health 2011 survey unless they had died, refused to participate in further surveys, or moved abroad.^20^

The plan and protocols of the Health 2000 and 2011 surveys were approved by the Ethics Committee for Research in Epidemiology and Public Health at the Hospital District of Helsinki and Uusimaa, and all protocols were conducted according to the Declaration of Helsinki. All participants gave written informed consent before participating in the surveys.^19^

Inclusion criteria for the present study are summarized in Figure 1. Those who did not participate in the health examination were excluded (n=1674). Those whose alcohol consumption exceeded 20 g/day for females and 30 g/day for males were excluded (n=739 at the baseline). As alcohol-associated liver disease is the leading cause of liver fibrosis alongside MASLD, and other causes for liver disease are rare, excluding those with excessive alcohol consumption is likely to generate a population with liver disease mostly attributable to metabolic dysfunction. Furthermore, participants who fasted <4 hours before blood sampling were excluded (n=155) since levels of serum collagen metabolites can be influenced by food intake. Those with missing values in even one of the baseline cognitive tests (n=130) and a missing ELF (n=215) were excluded. After these exclusions, 5115 participants were included in the cross-sectional analyses. Furthermore, those who did not take part in the Health 2011 survey or did not attend the cognitive assessment at the Health 2011 survey (n=2625) were not included in the longitudinal analyses. Thus, 2490 participants were included in the longitudinal analyses.

**FIGURE 1:**
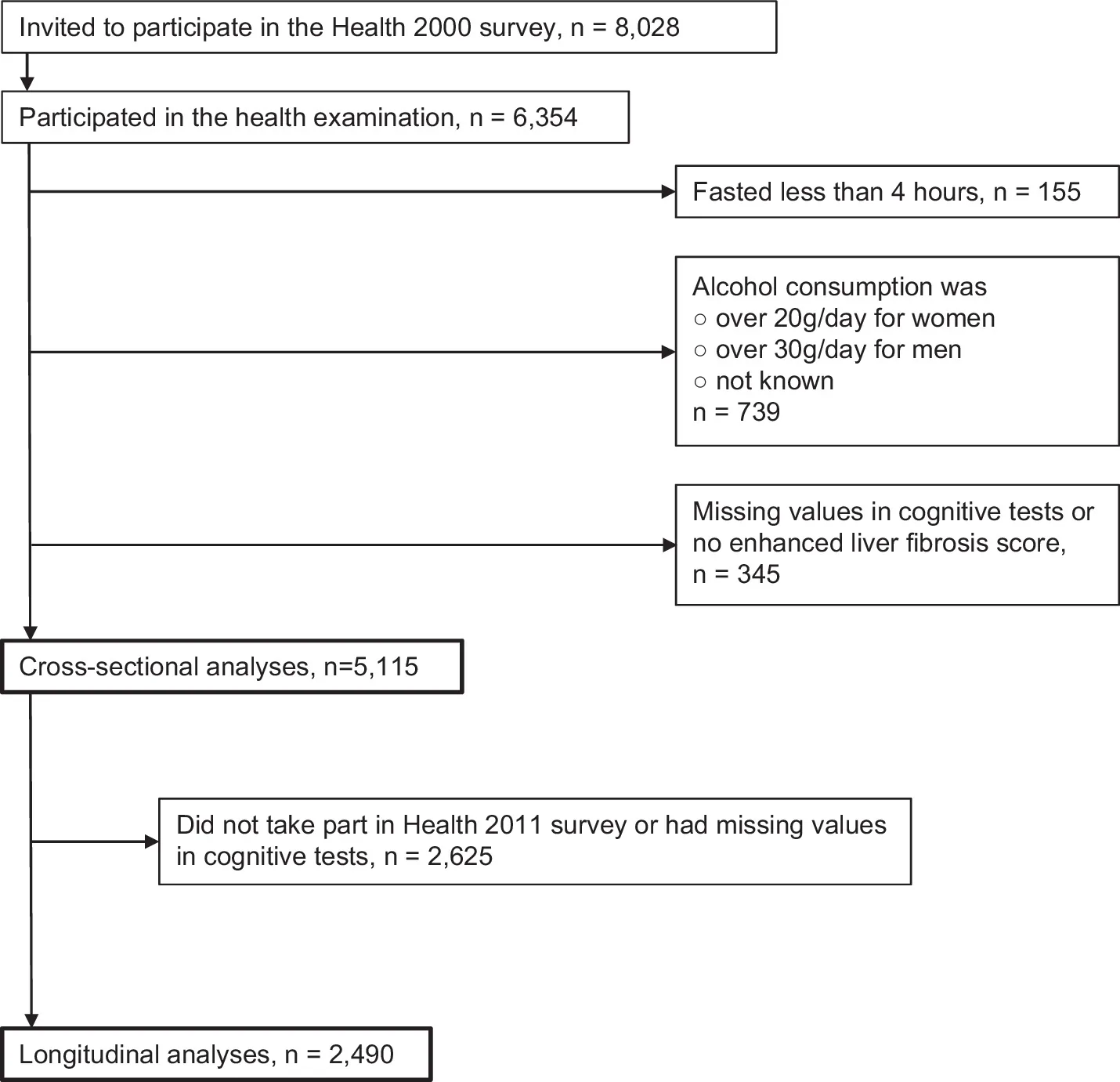
Flowchart of the study population.

### Enhanced liver fibrosis test (ELF)

ELF is a tool for predicting liver fibrosis based on 3 serum collagen metabolites: procollagen type III amino terminal propeptide (PIIINP), tissue inhibitor of metalloproteinases 1 (TIMP1), and hyaluronic acid (HA). The formula used to calculate the ELF was 2.278+0.851 ln(HA) +0.751 ln(PIIINP) +0.394 ln(TIMP1) (unit ng/mL).^9^

The metabolite concentrations were determined from frozen serum samples, stored at −70 °C, according to the instructions of the manufacturer (Siemens Healthcare Diagnostics Inc., Tarrytown, NY) using the ADVIA Centaur XPT analyzer (Siemens Healthcare Diagnostics Inc.).^9^

ELF has been shown to perform well in detecting liver fibrosis in patients with MASLD.^21^ The cutoff value used in this study for predicting moderate fibrosis was 9.8.^22^ The specificity of this cutoff has been estimated to be 98% for moderate fibrosis.^23^

### Cognitive tests

Cognitive tests for the Health 2000 and 2011 surveys were derived from the Consortium to Establish a Registry for Alzheimer’s Disease (CERAD) neurocognitive test battery. At both baseline and follow-up, participants underwent tests for verbal fluency (VF, language skills and executive functions), wordlist learning (WLL, verbal learning and memory), and delayed wordlist recall (DWLR, episodic memory). In addition, at baseline, simple reaction time (SRT) and multiple-choice reaction time (CRT) tests were conducted.

In the VF test, participants were asked to name as many animals as possible in 60 seconds.^19^ For the WLL test, participants were shown 10 words and asked to repeat them aloud and commit them to memory. After this, the participants were asked to list all the words they remembered. If the participant did not remember all the words in the first round, they were shown the words twice again but in different order, and once again asked to list all the words they remembered.^19^ For the DWLR test, the same list of words was used as for the WLL test, and participants were asked to repeat the same words after about 5 minutes, after SRT and CRT were conducted.^19^ In the SRT and CRT, a computer-based test was used (Good Response, Metitur Oy, Jyväskylä). In the test, the participants were to turn off a light from a switch with their dominant index finger as fast as they could when the light was turned on. A total of 4 lights were on the test panel, and during the SRT, the same light lit up 12 times at random intervals. In the CRT, all 4 lights lit up randomly 12 times. The SRT and CRT were conducted after 3 practice rounds, and after the participant had understood the test, and the result was accepted only if the response times were within the programmed time limits and they did not differ by more than 2 standard deviations from the participant's mean test score.^19^

### Covariates

Information on baseline education years, and physical activity levels were obtained from the study participants with a questionnaire.^20^ Alcohol consumption (g/L) was calculated based on a questionnaire asking about the frequency and amount of use of different alcoholic beverages.^20^ Physical activity was assessed by asking participants how often, in their free time, they exercised for at least 30 minutes and vigorously enough to cause sweating and mild breathlessness. The results were classified as: 1= a few times a year or less, 2= 2 or 3 times a month, 3= once a week, 4= 2 or 3 times a week, 5= 4–6 times a week, and 6= daily. Body mass index (BMI) was determined on the basis of measured weight and height by dividing weight (kg) by the square of height (m^2^).^20^ Blood pressure was measured twice in a sitting position from the right arm with a standard mercury manometer (Mercuro 300; Speidel & Keller, Jungingen, Germany), and the average of 2 measurements was used. Hypertension was defined as systolic blood pressure over 140 mm Hg or diastolic blood pressure over 90 mm Hg or the use of antihypertensive medication.^24^ From venous blood samples, total cholesterol, fasting glucose, insulin, and high-sensitivity-C-reactive protein (hs-CRP) were measured. Hypercholesterolemia was defined by having a serum cholesterol value over 6.5 mmol/L or the use of a lipid-lowering medication. DM was defined by having a fasting blood glucose value over 7 mmol/L or use of oral DM medication or insulin. Apolipoprotein E (APOE) genotyping was performed on those who gave consent for DNA sampling. IR was measured by calculating a homeostatic model assessment of insulin resistance (HOMA-IR) for each participant by multiplying fasting glucose (mmol/L) by fasting insulin (mU/L) and dividing by 22.5.^25^

### Statistical analysis

To analyze the effect of liver fibrosis on cognitive function, 2 groups were formed based on baseline ELF value (ELF<9.8 and ELF≥9.8). Mann–Whitney *U* test was used for continuous, non-normally distributed variables, Student *t* test for continuous, normally distributed variables, and χ^2^ test for categorical variables to evaluate differences between the groups.

Multivariable linear regression was used to examine the associations between (i) baseline ELF and baseline cognitive test scores, and (ii) baseline ELF and change in cognitive test scores during the follow-up period. The linear models were adjusted for covariates that are known to affect cognition based on the literature. The adjustment was conducted in 3 models: Model 1 was adjusted for age, sex, and education. Model 2 was adjusted additionally for APOE4 allele carriership, hypertension, hypercholesterolemia, DM, and BMI. Model 3 included all the variables in Model 2 and, in addition, smoking status, the Beck depression inventory score (BDI), and physical activity.

The effect of ELF was studied, as aforementioned, by dividing participants into 2 groups based on the ELF value (cutoff 9.8) at baseline. In addition, we also performed analyses where baseline ELF was treated as a continuous variable. To estimate an average ELF score during 11 years, the mean ELF score during the follow-up time was assessed by calculating the mean of the baseline and follow-up ELF scores.

We constructed a global cognitive z-score by combining the z-scores of VF, WLL, and DWLR to examine the association between ELF and overall cognition (Figure 2). The z-score of each cognitive test was calculated by standardizing the score of each individual to the mean and standard deviation of the baseline study population’s test scores. The composite z-score was then calculated as the mean of the individual tests’ z-scores. Lastly, the change in cognitive test scores was calculated by subtracting the baseline score from the follow-up cognitive test score, so that a lower score indicated greater decline. The associations between ELF and change in cognitive test scores were analyzed using the 3 models described previously. In addition to these adjustments, the baseline cognitive test score was adjusted for in all models.

**FIGURE 2:**
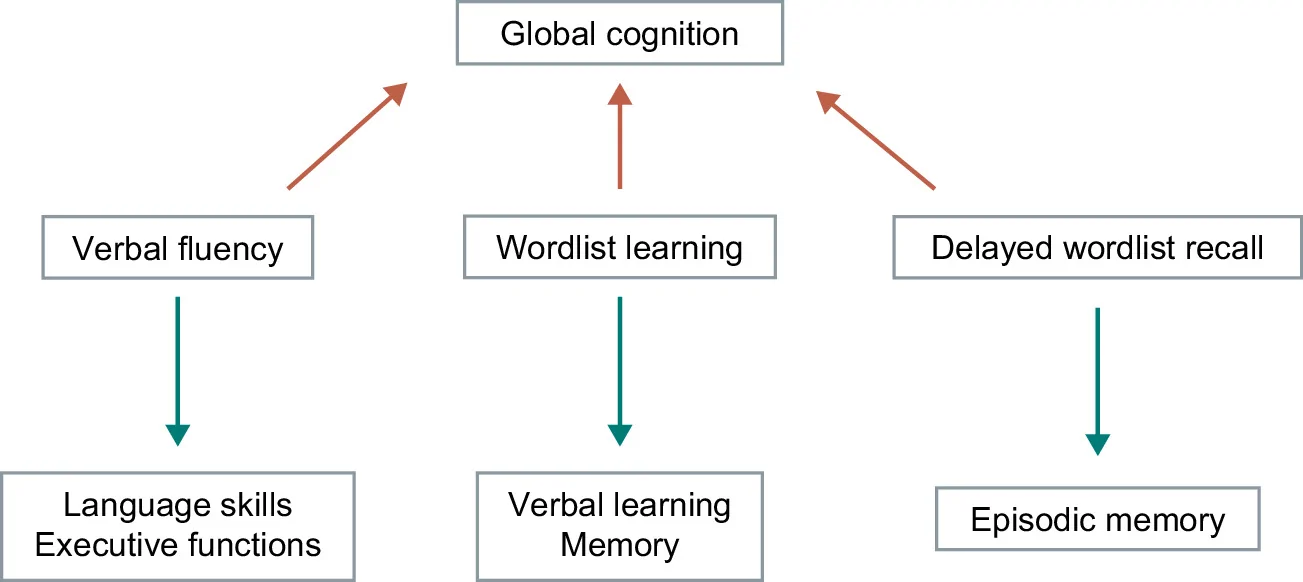
Cognitive domains included in the global cognitive z-score.

ELF, BDI, SRT, and CRT were skewed to the right, so a logarithmic transformation was used to achieve normal distribution. Other variables were normally distributed based on visual inspection. *p*=0.05 (two-tailed) was set as the level of significance. Statistical software used was SAS JMP Student Edition 18.2.1 (SAS Institute, Cary, NC, USA).

## RESULTS

### Characteristics of the study population

Detailed characteristics of the participants included in the longitudinal analyses can be found in Table 1, and of the cross-sectional analyses in Supplemental Table S1, https://links.lww.com/HC9/C496. Of the participants included in the cross-sectional analyses (n=5115), those with ELF <9.8 were younger, less likely to be females, more educated, smokers more frequently, and had a smaller BMI compared with the group with ELF ≥9.8. The prevalence of hypertension, hypercholesterolemia, and DM2 was higher among those with ELF ≥9.8. The frequency of APOE4 allele carriership was higher in the group with ELF <9.8. Both groups had similar physical activity levels and similar prevalence of sleep apnea. During the follow-up period, ELF increased more in those with ELF ≥9.8 (mean 0.7, SD 0.7) compared with the group with ELF <9.8 (mean 0.1, SD 0.5). For baseline cognitive test scores, those with ELF <9.8 performed better in every test compared with those with ELF ≥9.8. The participants included in longitudinal analyses had similar characteristics.

**TABLE 1:** Characteristics of the study participants who were included in the longitudinal analyses

	ELF<9.8 in 2000, N=2342	ELF≥9.8 in 2000, N=148	*p*-value ANOVA/χ^2^
Covariates in 2000			
Age, years, N=2490	47.7 (11.2)	63.1 (8.7)	<0.0001
Sex, number of females (%), N=2490	1362 (58.2)	82 (55.4)	0.5
Education, years, N=2475	12.3 (3.8)	9.8 (3.5)	<0.0001
BMI (kg/m^2^), N=2489	26.2 (4.2)	28.9 (4.2)	<0.0001
Smoking, n (%), N=2481	515 (22.1)	14 (9.5)	<0.0001
Hypertension, n (%), N=2478	853 (36.6)	92 (62.2)	<0.0001
Hypercholesterolemia, n (%), N=2488	557 (23.8)	56 (38.1)	0.0002
Diabetes mellitus, n (%), N=2488	67 (2.9)	13 (8.8)	0.0006
GGT (U/L), N=2490	29.5 (26.9)	38.1 (74.8)	0.2
Hs-CRP (mg/L), N=2488	1.6 (3.6)	2.8 (5.1)	0.003
HOMA-IR (mmol/L × mU/L), N=2488	1.9 (1.6)	2.5 (2.1)	0.0002
APOE4 carriership, n of carriers (%), N=2407	726 (32.0)	39 (27.9)	0.3
Beck depression inventory (BDI) score, N=2470	6.0 (6.2)	8.9 (7.6)	<0.0001
Physical activity, lower score indicates more exercise, N=2470	3.0 (0.9)	3.0 (0.9)	0.8
Sleep apnea, n (%), N=2354	25 (1.1)	2 (1.4)	0.7
Cognitive tests in 2000			
Verbal fluency score, N=2490	25.4 (6.8)	24.3 (6.6)	0.06
Wordlist learning score, N=2490	22.1 (3.7)	20.0 (3.8)	<0.0001
Delayed wordlist recall score, N=2490	7.6 (1.7)	6.7 (1.8)	<0.0001
Global cognitive z-score, N=2490	0.9 (2.0)	−0.2 (2.1)	<0.0001
Simple reaction time (ms), N=2437	0.3 (0.06)	0.3 (0.07)	0.02
Multiple-choice reaction time (ms), N=2406	0.4 (0.1)	0.5 (0.1)	<0.0001
Cognitive tests in 2011			
Verbal fluency score, N=2490	24.7 (7.2)	21.2 (6.3)	<0.0001
Wordlist learning score, N=2490	21.8 (4.1)	18.3 (4.1)	<0.0001
Delayed wordlist recall score, N=2490	7.5 (2.0)	6.0 (2.1)	<0.0001
Global cognitive z-score, N=2490	0.1 (2.5)	−1.9 (2.5)	<0.0001
Change in cognitive test scores			
Change in verbal fluency, N=2490	−0.6 (6.0)	−3.1 (5.7)	<0.0001
Change in wordlist learning, N=2490	−0.3 (3.4)	−1.7 (3.8)	<0.0001
Change in delayed wordlist recall, N=2490	−0.1 (1.6)	−0.7 (1.8)	0.0001
Change in global cognitive z-score, N=2490	−0.8 (1.7)	−1.8 (1.9)	<0.0001

*Note:* The results are shown as mean (standard deviation) unless stated otherwise. For continuous non-normally distributed variables, the Mann–Whitney *U* test and for continuous normally distributed variables, the Student *t* test were used to examine differences between the groups. χ^2^ test was used for categorical variables.

Abbreviations: ANOVA, analysis of variance; APOE, apolipoprotein E; BDI, Beck depression inventory score; BMI, body mass index; ELF, enhanced liver fibrosis; GGT, gamma-glutamyl transferase; HOMA-IR, homeostatic model assessment of insulin resistance.

Those who were not included in the baseline study population (n=2913) due to not attending the health examination proper in 2000–2001, missing ELF or cognitive test scores, or excessive alcohol consumption, were more likely to be male than female (52.2% vs. 47.8%, *p*<0.0001), older (56.1 vs. 53.2 y, *p*<0.0001), and less educated (10.5 vs. 11.1 y, *p*<0.0001) than those who were included.

### Cross-sectional associations between ELF and cognitive performance at baseline

Estimated mean values (95% confidence intervals) from cross-sectional analyses are shown in Table 2. ELF ≥9.8 was independently associated cross-sectionally at baseline with poorer global cognitive z-score, WLL, SRT, and CRT in the fully adjusted Model 3. In contrast, VF and DWLR did not differ between the ELF groups in any of the models.

**TABLE 2:** Cross-sectional analyses on the effect of ELF on cognitive performance in 2000

	ELF<9.8, N=4413	ELF≥9.8, N=702	*p*
Model 1, N=5070			
Global cognitive z-score	−0.02 (−0.08 to 0.04)	−0.3 (−0.4 to −0.1)	**0.004**
Verbal fluency	23.6 (23.4–23.8)	23.1 (22.5–23.6)	0.09
Wordlist learning	20.5 (20.4–20.6)	19.9 (19.6–20.2)	**0.0002**
Delayed wordlist recall	7.0 (6.9–7.0)	6.8 (6.7–7.0)	0.1
Simple reaction time	−1.14 (−1.15 to −1.13)	−1.10 (−1.12 to −1.08)	**0.0001**
Multiple-choice reaction time	−0.77 (−0.78 to −0.76)	−0.74 (−0.76 to −0.72)	**0.006**
Model 2, N=4878			
Global cognitive z-score	−0.2 (−0.3 to −0.03)	−0.4 (−0.6 to −0.2)	**0.009**
Verbal fluency	23.2 (22.7–23.6)	22.8 (22.1–23.4)	0.2
Wordlist learning	20.4 (20.1–20.6)	19.7 (19.4–20.1)	**0.0004**
Delayed wordlist recall	6.9 (6.8–7.0)	6.8 (6.6–6.9)	0.2
Simple reaction time	−1.12 (−1.13 to −1.10)	−1.08 (−1.11 to −1.06)	**0.0011**
Multiple-choice reaction time	−0.75 (−0.76 to −0.73)	−0.72 (−0.75 to −0.70)	**0.02**
Model 3, N=4061			
Global cognitive z-score	−0.2 (−0.3 to −0.03)	−0.4 (−0.6 to −0.2)	**0.02**
Verbal fluency	23.2 (22.8–23.7)	22.9 (22.1–23.6)	0.3
Wordlist learning	20.3 (20.0–20.5)	19.7 (19.3–20.1)	**0.003**
Delayed wordlist recall	6.9 (6.7–7.0)	6.7 (6.6–6.9)	0.2
Simple reaction time	−1.12 (−1.14 to −1.10)	−1.08 (−1.11 to −1.06)	**0.0009**
Multiple-choice reaction time	−0.75 (−0.76 to −0.73)	−0.72 (−0.75 to −0.70)	**0.02**

*Note:* Results are shown as adjusted mean values (95% confidence intervals). Model 1 was adjusted for age, sex, and education. Model 2 was adjusted for Model 1+hypercholesterolemia, hypertension, diabetes mellitus, BMI, and APOE4 carriership. Model 3 was adjusted for Model 2+smoking status, physical activity, and BDI.

Abbreviations: APOE, apolipoprotein E ε4; BDI, Beck depression inventory; BMI, body mass index; ELF, enhanced liver fibrosis.

When treating ELF as a continuous variable, ELF was not cross-sectionally associated with global cognitive z-score. ELF was inversely associated with WLL in Models 1 and 2. In Model 3, the association was lost. Continuous ELF was not cross-sectionally associated with VF, DWLR, SRT, or CRT.

### Longitudinal associations between ELF and cognitive performance

Next, we studied how categorical ELF was associated with a change in cognitive test scores between baseline and follow-up (Table 3). A larger decline in global cognitive z-score and WLL was observed for those with ELF ≥9.8 compared with ELF <9.8, independently of confounding factors. Those with ELF ≥9.8 exhibited a larger decline in DWLR test scores before adjusting for covariates in Model 3.

**TABLE 3:** Associations between ELF groups and change in cognitive test scores during the follow-up period from 2000 to 2011

	ELF<9.8 in 2000, N=2342	ELF≥9.8 in 2000, N=148	*p*-value independent *t* test
Model 1, N=2475			
Global cognitive z-score	−0.8 (−0.9 to −0.8)	−1.2 (−1.5 to −1.0)	**0.002**
Verbal fluency	−0.8 (−1.0 to −0.5)	−1.6 (−2.5 to −0.7)	0.09
Wordlist learning	−0.4 (−0.6 to −0.3)	−1.2 (−1.7 to −0.7)	**0.005**
Delayed wordlist recall	−0.1 (−0.2 to −0.09)	−0.4 (−0.7 to −0.2)	**0.03**
Model 2, N=2386			
Global cognitive z-score	−0.8 (−1.0 to −0.5)	−1.2 (−1.5 to −0.8)	**0.003**
Verbal fluency	−0.4 (−1.1 to 0.4)	−1.2 (−2.4 to −0.06)	0.09
Wordlist learning	−0.4 (−0.9 to 0.008)	−1.2 (−1.8 to −0.5)	**0.006**
Delayed wordlist recall	−0.1 (−0.3 to 0.1)	−0.4 (−0.7 to −0.08)	**0.03**
Model 3, N=2006			
Global cognitive z-score	−0.9 (−1.1 to −0.6)	−1.3 (−1.6 to −0.9)	**0.01**
Verbal fluency	−0.6 (−1.5 to 0.2)	−1.4 (−2.7 to −0.2)	0.1
Wordlist learning	−0.7 (−1.2 to −0.2)	−1.4 (−2.1 to −0.7)	**0.02**
Delayed wordlist recall	−0.2 (−0.4 to 0.03)	−0.5 (−0.8 to −0.1)	0.08

*Note:* Results are shown as adjusted mean values (95% confidence intervals). Model 1 was adjusted for baseline age, sex, and education. Model 2 was adjusted for Model 1+ baseline hypercholesterolemia, hypertension, diabetes mellitus, BMI, and APOE4 carriership. Model 3 was adjusted for Model 2+ baseline smoking status, physical activity, and BDI. All models were additionally adjusted for baseline cognitive scores.

Abbreviations: APOE, apolipoprotein E ε4; BDI, Beck depression inventory; BMI, body mass index; ELF, enhanced liver fibrosis.

ELF as a continuous variable did not associate with global cognitive z-score. It was inversely associated with a decline in WLL in all models but did not associate with VF or DWLR. Similarly, ELF was inversely associated with change in WLL but not with change in VF, DWLR, or global cognitive z-score.

### Association between mean ELF across baseline and follow-up and cognitive test scores at follow-up

The continuous mean value of baseline and follow-up ELF predicted poorer global cognitive z-score in Models 1 and 3, but in Model 2, the *p*-value rose to 0.053 (Table 4). Mean ELF was associated with worse WLL at follow-up, independently of confounding factors. In addition, mean ELF was associated with poorer DWLR in Model 1, but the association weakened after adjusting for covariates in Model 2. Mean ELF did not predict follow-up VF.

**TABLE 4:** Associations between mean ELF and follow-up test scores

	Mean ELF	*p*
Model 1, N=2475		
Global cognitive z-score	−1.5 (0.7)	**0.03**
Verbal fluency	−0.3 (2.3)	0.9
Wordlist learning	−3.8 (1.2)	**0.002**
Delayed wordlist recall	−1.2 (0.6)	**0.047**
Model 2, N=2386		
Global cognitive z-score	−1.4 (0.7)	0.053
Verbal fluency	−0.5 (2.5)	0.98
Wordlist learning	−3.8 (1.3)	**0.003**
Delayed wordlist recall	−1.1 (0.6)	0.09
Model 3, N=2006		
Global cognitive z-score	−1.6 (0.8)	**0.04**
Verbal fluency	−1.3 (2.7)	0.6
Wordlist learning	−3.6 (1.4)	**0.009**
Delayed wordlist recall	−1.2 (0.7)	0.09

*Note:* Results are shown as estimates (standard error). Mean ELF was calculated as the sum of ELF in 2000 and 2011 divided by 2. Model 1 was adjusted for age, sex, and education. Model 2 was adjusted for Model 1+hypercholesterolemia, hypertension, diabetes mellitus, BMI, and APOE4 carriership. Model 3 was adjusted for Model 2+smoking status, physical activity, and BDI.

Abbreviations: APOE, apolipoprotein E ε4; BDI, Beck depression inventory; BMI, body mass index; ELF, enhanced liver fibrosis.

The association between the mean ELF and the change in cognitive function was also examined. Mean ELF was associated with a change in global cognitive z-score in Model 1 and Model 2, but the association diminished after further adjusting. A higher mean ELF was independently associated with a greater decline in WLL. Mean ELF was not associated with a change in VF nor DLWR.

## DISCUSSION

In this study, we show for the first time that liver fibrosis, measured by ELF, is independently associated with poorer global cognition both cross-sectionally and longitudinally, as well as greater decline in global cognition over an 11-year follow-up, in a nationally representative sample of the Finnish adult population. Based on multivariable regression adjusted for age and other confounders, liver fibrosis is cross-sectionally associated with poorer WLL, and slower SRT and CRT. In addition, liver fibrosis is longitudinally associated with poorer WLL and predicts a greater decline in WLL during the 11-year follow-up.

Individuals with elevated ELF were older and less educated compared with those with lower ELF (Table 1), and these factors are likely to contribute to the differences noted between high-ELF and low-ELF groups. Thus, adjustments were made in multivariable linear regression to address these potential confounding effects. For global cognitive score, WLL, SRT, and CRT, the associations remained after adjusting in Models 1–3, but were lost for VF and DWLR.

Others have previously studied the effect of liver fibrosis on cognition in patients with MASLD, but unlike in our study, the sample sizes have been relatively small, and these studies lacked longitudinal analyses. Cross-sectionally, non-alcoholic steatohepatitis was associated with poorer cognitive performance in a Thai sample of 319 individuals with HIV infection from the HIV-NAT 207/Aging study.^18^ Liver fibrosis was associated with microvascular brain pathology and cognitive impairment in a Dutch sample of 156 individuals of the BARICO study.^26^ Furthermore, liver fibrosis, measured with the Fibrosis-4 score, was associated with faster decline in performance in the Montreal Cognitive Assessment (MoCA) score, the Symbol Digit Modalities, Benton Judgment of Line Orientation, and the Modified Semantic Fluency tests in a cohort of 409 patients with Parkinson disease.^27^ Moreover, high risk for fibrosis, measured by NAFLD fibrosis score (NFS), was associated with poorer performance in executive functions and abstract reasoning in a subpopulation of the Framingham study, even though MASLD did not associate with poorer cognitive function.^13^

Minimal hepatic encephalopathy is commonly seen in patients with cirrhosis. It is thought to be a consequence of altered nitrogen metabolism^28^ and peripheral inflammation.^29^ In minimal hepatic encephalopathy, deficits in attention, working memory, response inhibition, and executive function are detected.^30^ In this study, WLL measures working memory and attention, and SRT measures executive function. The similarities may reflect shared mechanisms of the liver–brain axis, since the subjects of our study did not have cirrhosis and thus did not meet the criteria for minimal hepatic encephalopathy.

In addition to minimal hepatic encephalopathy, possible mechanisms behind the association between ELF and decline in attention and working memory in this study could be insulin resistance, low-grade inflammation, dysregulated metabolism of lipids and free fatty acids, psychiatric diseases, and cardiovascular disease^31^ as is shown by increased HOMA-IR, hs-CRP, and BDI score and higher incidence of DM2, hypertension and hypercholesterolemia among the individuals with ELF ≥9.8. However, many of these factors are intertwined and contribute to cognitive decline independently. Thus, it is difficult to pinpoint the causal factor that drives cognitive decline in liver fibrosis patients.

The strengths of this study were the large nationally representative sample of the Finnish adult population, the 11-year follow-up period with repeated cognitive tests, and a vast selection of confounding factors. However, the study also had limitations. Firstly, SRT and CRT were not repeated in the follow-up examination. Secondly, a transient elastography or biopsy-proven liver fibrosis would have provided more reliable results, as ELF is a non-diagnostic tool and may give false positives.^32^ Furthermore, in the longitudinal analyses, Model 3 only had 148 participants with ELF ≥9.8, and thus, there is a possibility of overfitting. However, the adjusted mean value is similar to those of the less-adjusted Models 1 and 2.

This study demonstrated for the first time, to our knowledge, that liver fibrosis, measured by ELF, is longitudinally associated with a decline in verbal learning, attention, and working memory. These findings are of importance considering the aging population and increasing prevalence of MASLD. This suggests a future increase in cognitive impairment, posing a challenge for public health. Therefore, it is important to develop widely available, specific, and sensitive screening and diagnostic tools for the early detection of MASLD, as well as efficient prophylactic actions and treatment options.

## Supplementary Material
