# Supplementary material for: Enhanced liver fibrosis score is associated with cognitive performance during an 11-year follow-up period
Source: Hepatol Commun. 2026 Oct 19;10(11):e1059. doi: 10.1097/HC9.0000000000001059 (PMC13682807; doi:10.1097/HC9.0000000000001059)
Supplement: Supplementary file 1 [file hc9-10-e1059-s001.docx]

Table S1. Characteristics of the participants included in the baseline cross-sectional analyses.

|  | ELF < 9.8 | ELF > 9.8 | P value |
| --- | --- | --- | --- |
| Age, years  N=5,115 | 50.1 (13.3) | 72.2 (11.3) | <.0001 |
| Sex, female (%)  N=5,115 | 2,559 (58.0) | 438 (62.4) | 0.03 |
| Education, years  N=5,070 | 11.6 (4.0) | 8.1 (3.5) | <.0001 |
| Body mass index  N=5,111 | 26.6 (4.5) | 28.5 (4.9) | <.0001 |
| BDI, score  N=4,981 | 6.5 (6.6) | 9.8 (7.1) | <.0001 |
| Hypertension, number (%)  N=5,101 | 1,898 (43.1) | 509 (72.6) | <.0001 |
| Diabetes mellitus, number (%)  N=5,113 | 144 (3.3) | 96 (13.7) | <.0001 |
| Hypercholesterolemia, number (%)  N=5,113 | 1,123 (25.5) | 232 (33.1) | <.0001 |
| Physical activity, higher score indicates more exercise  N=4,980 | 3.0 (0.9) | 3.1 (0.9) | 0.1 |
| Smoking status, number  N=5,097 | 1,110 (25.2) | 64 (9.2) | <.0001 |
| GGT, U/l  N=5,115 | 30.9 (31.0) | 42.2 (69.1) | <.0001 |
| APOE4 allele carriership, number (%)  N=4,930 | 1,371 (32.2) | 180 (26.9) | 0.006 |
| Hs-CRP, mg/l  N=5,110 | 1.9 (5.5) | 3.7 (9.0) | <.0001 |
| HbA1c, %  N=5,106 | 5.3 (0.5) | 5.6 (0.8) | <.0001 |
| HOMA-IR  N=5,113 | 2.1 (2.4) | 3.2 (4.1) | <.0001 |
| Cognitive tests in 2000 | | | |
| Verbal fluency, score  N=5,115 | 24.1 (7.1) | 20.0 (6.7) | <.0001 |
| Wordlist learning, score  N=5,115 | 21.1 (4.3) | 17.0 (4.9) | <.0001 |
| Delayed wordlist recall, score  N=5,115 | 7.2 (1.9) | 5.5 (2.2) | <.0001 |
| Global cognitive Z-score  N=5,115 | 0.3 (2.4) | -2.0 (2.6) | <.0001 |
| Simple reaction time, ms  N=4,857 | 0.32 (0.077) | 0.38 (0.0034) | <.0001 |
| Choice reaction time, ms  N=4,706 | 0.47 (0.12) | 0.58 (0.0056) | <.0001 |

The results are shown as mean values (standard deviation) unless stated otherwise. For continuous non-normally distributed variables, Mann-Whitney U test and for continuous normally distributed variables, Student’s t-test was used to examine differences between the groups. Chi-square test was used for categorical variables. Abbreviations: APOE4=apolipoprotein E4, BDI=Beck’s depression inventory score, ELF=enhanced liver fibrosis, GGT=gamma glutamyl transferase, HOMA-IR=homeostatic model assessment of insulin resistance.
